# A Single-Cell Omics Technical Guide for Advancing Neuropsychiatric Research

**DOI:** 10.3390/genes16121394

**Published:** 2025-11-21

**Authors:** Kayleigh Casmey, Maria Zimmermann, Yuxin Xie, Sierra A. Codeluppi-Arrowsmith, Gustavo Turecki

**Affiliations:** 1 McGill Group for Suicide Studies, Douglas Mental Health University Institute, Montreal, QC H4H 1R3, Canada; kayleigh.casmey@mail.mcgill.ca (K.C.); maria.zimmermann@mail.mcgill.ca (M.Z.); yuxin.xie@mail.mcgill.ca (Y.X.); sierra.codeluppi@mail.mcgill.ca (S.A.C.-A.); 2 Integrated Program in Neuroscience, McGill University, Montreal, QC H3A 1A1, Canada; 3 Department of Human Genetics, McGill University, Montreal, QC H3A 1Y2, Canada; 4 Department of Psychiatry, McGill University, Montreal, QC H3A 1A1, Canada

**Keywords:** single-cell omics, neuropsychiatric disorders, human brain

## Abstract

Single-cell omics technology has advanced rapidly since its inception, offering increasing precision, resolution, and technical diversity to explore cell-specific molecular features in the human brain and neuropsychiatric disorders. While traditional bulk genomic analyses have provided valuable insights into the molecular processes of these disorders, single-cell omics allows for the investigation of cellular heterogeneity in the brain, which is crucial for dissecting underlying pathology. Neuropsychiatric disorders—such as dementia and depression—are complex and heterogenous brain disorders driven by intricate interactions of genetic and environmental factors. Methodological developments in single-cell omic technologies have enabled their application directly to human brain tissue for the study of neuropsychiatric disorders, yielding cell-specific insights in transcriptomics and epigenomics, with emerging findings in proteomics, metabolomics, multi-omics, and beyond. This review discusses different single-cell omic technologies, focusing on their application to postmortem human brain tissue, highlighting key findings from the use of these methods in neuropsychiatric disorders, and providing considerations for future implementation to elucidate the molecular landscape of brain changes associated with these conditions.

## 1. Introduction

The human body contains trillions of cells, each having effectively the same genotype yet giving rise to incredibly varied phenotypes to form distinct tissues and organs. The complexity, dimensionality, and specificity of molecules—DNA, RNA, proteins, and their modifications—shape cell identity and function. The human brain is composed of billions of cells that are highly diverse and organized to form specialized structures and orchestrate intricate functions like perception, cognition, and emotion. When such brain functions are impaired in neuropsychiatric disorders, what changes occur at the cellular and molecular level? Until recently, studies addressing these questions typically relied on brain tissue homogenates, which dilute molecular signals, obscure cell-specific alterations, and can introduce bias when comparing groups if tissue composition differs. The development of single-cell omic techniques has not only overcome these limitations but has also enabled major advances in our understanding of the human brain.

**Figure 1:**
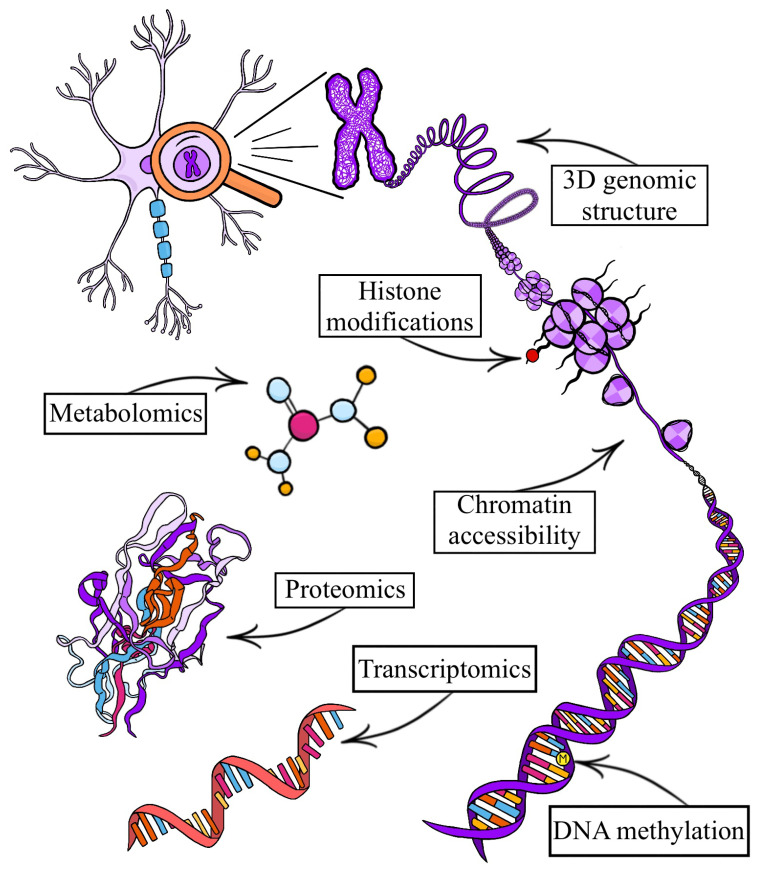
Aspects of molecular exploration using single-cell omics.

Single-cell omics simultaneously profiles the dynamic molecular architecture of thousands of individual cells with exceptional granularity (Figure 1). The advancement of *spatially resolved* single-cell omic technologies has further provided essential context to cells by mapping molecular features within tissues. This molecularly detailed picture of the cell can ultimately deepen our understanding of neuropsychiatric disorders to advance efforts in prediction, prevention, diagnosis, and personalized therapeutic approaches. The precision, resolution, and technical diversity of these technologies have already provided cell-specific insights into human brain functions, development, aging, biomarkers, and mechanisms of disorders [1,2]. Notably, there are now a plethora of human brain atlases and other open access resources for single-cell omics, which are instrumental for the study of the brain broadly (Table 1). Clinical applications of single-cell omics have not yet been well established, but progress is being made towards using these technologies to help guide clinical practice [2]. Particularly, single-cell omics can be used in drug discovery and screening to improve therapeutic efficacy, predict drug response and targets, and facilitate personalized treatments [3,4]. Single-cell omics technology continues to expand in various directions, including the combination of multiple omic methods to profile individual cells (i.e., single-cell multi-omics), integration of other experimental techniques (e.g., long-read sequencing) with single-cell omics, and the emergence of more types of single-cell omic exploration (e.g., single-cell metabolomics).

Previous review papers have summarized single-cell omic approaches from diverse perspectives, including the underlying principles, methodologies, technical considerations, and corresponding bioinformatic and statistical approaches that are integral for analysis and visualization of single-cell omic data [1,5,6,7,8,9,10,11,12,13,14,15,16,17,18,19,20,21,22,23,24,25,26,27,28,29]. As this review focuses on experimental single-cell omic techniques, computational methods are outside the scope and have been reviewed in detail elsewhere [30,31,32,33,34,35,36,37]. The aim of this review is to provide a fundamental technical guide and roadmap for applying single-cell and spatial omic technologies in archival, postmortem human brain tissue for the investigation of neuropsychiatric disorders. This technology-centered review informs and inspires future neuropsychiatric research using single-cell omics by tracing the development of these techniques, discussing methodologies and applications in postmortem human brain tissue, highlighting current uses and advancements in neuropsychiatric research, and proposing how emerging platforms could be leveraged in future studies. While this is not a systematic review, our methods included the identification of papers through broad search terms (e.g., single-cell omic, spatially resolved, and postmortem human brain) in PubMed and Google Scholar. Additional papers were found by scanning the references of these papers. We included all original papers using single-cell omics in postmortem human brain that were identified before October 2025.

**Table 1:** Open access single-cell omic resources for the study of the human brain (All accessed on 7 November 2025).

Name	Website	Citation
CZ CellxGene Discovery	https://cellxgene.cziscience.com	[38]
Human Cell Atlas	https://data.humancellatlas.org	[39]
Brain Cell Atlas	https://brain.ifr.unifiedcellatlas.org:61011/#/index	[40]
Cis-element Atlas (CATlas)	https://catlas.org/catlas/	[41]
Single Cell Portal	https://singlecell.broadinstitute.org/single_cell	[42]
Singlocell	https://singlocell.openscience.mcgill.ca/	
PsychENCODE	https://www.psychencode.org/	[43]
ENCODE	https://www.encodeproject.org/	[44]
Allen Brain Cell (ABC) Atlas	https://portal.brain-map.org/atlases-and-data/bkp/abc-atlas	[45]
BrainScope	https://brainscope.app	[46]
BRAIN Initiative Cell Census Network (BICCN)	https://biccn.org/	[47]
BRAIN Initiative Cell Atlas Network (BICAN)	https://www.portal.brain-bican.org	
SpatialLIBD	https://research.libd.org/spatialLIBD/	[48,49]
UCSC Cell Browser	https://cells.ucsc.edu/	[50]
The Human Protein Atlas	https://www.proteinatlas.org/humanproteome/single+cell	[51]
Spatio-temporal Cell Atlas of the Human Brain (STAB)	https://mai.fudan.edu.cn/stab/explore/cell-subtype/cell_subtype	[52]
Multi-omics Atlas of the Primate Brain (MAPbrain)	http://bigdata.ibp.ac.cn/mapBRAIN/	[53]
Spatial Transcript Omics DataBase (STOmics DB)	https://db.cngb.org/stomics/	[54]

## 2. Technological Advancements That Led to Single-Cell Omics

Over the past couple decades, single-cell omics has evolved through a series of methodological innovations aimed at achieving high-throughput analysis of molecular content from many individual cells. This was preceded by the development of several foundational molecular biology techniques. Previously, studies were limited by the low amount of molecular material within one cell, and techniques such as polymerase chain reaction (PCR) [55] and in vitro transcription (IVT) [56] were developed to amplify nucleic acids for reliable detection and quantification. In 1990, Brady et al. [57] used poly(dT) primers to amplify cDNA from individual macrophage-like mouse cells using PCR. Then, in 1992, Eberwine et al. [58] applied IVT to quantify select transcripts from single, live rat hippocampal neurons. Although these studies demonstrated that gene expression could be measured at the level of an individual cell, early omic assays in single cells were limited by low cellular and molecular throughput.

In 2009, Tang et al. [59] established an mRNA sequencing assay to generate sufficient material for whole-transcriptome analysis at the single-cell level. This landmark single-cell study used PCR-based amplification and next-generation sequencing (NGS) to profile RNA from manually picked mouse blastomeres [59]. They were able to detect 75% more genes than microarrays, discover previously unknown splice junctions, and reveal transcript isoforms within the same cell [59]. This single-cell study was foundational for the field, as it revealed gene expression heterogeneity within individual cells, thus underscoring the importance of single-cell investigation [59].

A particular challenge for Tang and colleagues’ [59] early single-cell omic study, and for the field broadly, was the isolation of individual cells. Early single-cell studies relied on manual isolation, in which individual cells were separated using a microscope and micropipette [59]. These methods are labour-intensive, have low cellular throughput, and are restricted to large, morphologically identifiable cells. The implementation of fluorescence-activated cell sorting (FACS), a now widely used flow cytometry-based sorting method, has greatly facilitated the process of single-cell isolation [60]. This technology was also adapted for the isolation of nuclei, named fluorescence-activated nuclei sorting (FANS) [61]. FACS/FANS is a semi-automated technique that can be used to discriminate cells/nuclei from debris and isolate specific populations of cells/nuclei (e.g., neurons) [62]. FACS/FANS applies high-frequency oscillations to separate cells/nuclei from a stream of liquid into individual droplets [63]. Cells/nuclei that have been incubated with fluorophore-conjugated antibodies (e.g., anti-NeuN, which targets neuronal nuclei) or stained by fluorescent dyes (e.g., Hoechst) can be identified using a laser and detector system [62,63]. The individual droplets containing a cell/nucleus of interest are electrically charged, and deflection plates are used to sort them into separate tubes/individual wells [63]. This provides a high-purity isolation of cells/nuclei and greatly improves scalability compared to manual picking [62,63]. Another early key development for isolating individual cells was magnetic-activated cell sorting (MACS), which allows large-scale cell sorting according to specific markers on the cell membrane that bind to magnetic beads [64]. Cells are passed through a high-gradient magnetic column, and those that are unbound pass through, while magnetic bead-bound cells are retained and afterward eluted [64]. Finally, a major technological advancement for the isolation of single cells was microfluidic technology. Microfluidic devices first emerged for chemical and engineering applications but were adapted to biology in the early 2010s for capturing and processing individual cells in miniaturized reaction chambers [65]. These devices have microfluidic channels that precisely manipulate small volumes of fluid to isolate individual cells by encapsulating them in droplets within an oil emulsion.

## 3. Foundations of Single-Cell Omics: Single-Cell Transcriptomics and Core Concepts

Single-cell transcriptomics, the first single-cell high-throughput omic technique and Nature’s method of the year in 2013, has advanced considerably since the first single-cell mRNA-seq study [59,66]. This approach profiles thousands of RNAs, primarily messenger RNAs (mRNA), from thousands of individual cells in parallel, thus providing cell-specific gene expression profiles to unravel heterogeneous cell populations. Many methodological advancements in single-cell transcriptomics have been made over the years, such as the implementation of cell-specific barcodes that are unique, random or semi-random, non-coding nucleic acid sequences that hybridize to target molecules [67,68,69,70]. Cell barcoding is an efficient way to acquire molecular information from thousands of cells in parallel and subsequently map molecules to their respective cells, thereby increasing processing capacity and reducing experimental costs [67,68,69,70]. Similarly, the introduction of labeling individual input molecules with random sequences called unique molecular identifiers (UMIs) reduces PCR amplification noise and improves overall data quality [71]. Additionally, the application of single-cell transcriptomics has been expanded to various tissues—most pertinent to this review, the human brain [72,73,74,75,76,77,78,79,80,81,82]. This rapid technological progress has resulted in many significant findings in the human brain over a short period of time. These findings include the identification and characterization of brain cell types and subtypes, molecular insights into the developing brain, comparative animal studies that have revealed species-specific features, and the association of certain cell types and genes with disorders [40,72,73,76,77,80,82,83,84,85,86,87,88,89,90].

### 3.1. Single-Cell or Single-Nucleus?

Single-cell RNA sequencing (scRNA-seq) and single-nucleus RNA sequencing (snRNA-seq) both profile RNA, either in whole cells or nuclei isolated from cells. Determining whether to use cells or nuclei depends largely on the research being carried out and, particularly, input material. Commonly used tissue dissociation methods to isolate cells for scRNA-seq can often be too harsh for many tissue types, including the brain, which can damage cells and alter transcription profiles [91,92]. Some cell types may be more susceptible to damage during tissue dissociation, particularly neurons, as they are highly interconnected, thus leading to artificial selection of cells and a skewed representation of cell types [75,91,93]. An additional consideration when studying human brain tissue is the difficulty in obtaining samples. As a result, postmortem brain samples have become an invaluable means for research. Preserved human brain tissue is often fresh-frozen (FF), fixed fresh-frozen (FxF), or formalin-fixed paraffin-embedded (FFPE). Samples used for single-cell transcriptomics in human brain often come from fresh-frozen postmortem tissue. This tissue can be of poor quality from inherent constraints around preservation (e.g., postmortem interval), and it is difficult to isolate intact cells from frozen tissue, as the freeze–thaw process damages cell membranes [94,95]. The advantage of snRNA-seq is that nuclei are more resistant than cells to freeze–thaw stresses, and nuclear transcriptomes have been shown to provide comparable results in expression to whole cell transcriptomes, showing their ability to effectively profile cell type expression dynamics [91,96]. However, nuclear transcriptomes are not fully equivalent to whole-cell transcriptomes. For example, the analysis of nuclei lacks information from cytoplasmic RNA, which can be important for the investigation of particular cell types, like for profiling microglial activation genes [97]. This is expressly important for neurons, as nuclear information may not reflect molecular processes occurring in different cellular subcompartments, such as dendrites. Additionally, RNA of dissociated cells in nuclei suspensions (i.e., ambient RNA) can interfere with single-cell assessment and cause masking or misinterpretation of cell identity [98]. Technological solutions that have addressed such limitations will be discussed throughout the review. See Butto et al. [9] for an in-depth review on the use of nuclei for NGS. The term single-cell is used throughout this review, and in the field broadly, to refer to the analysis of individual cells, regardless of whether the input material is cells or nuclei. When relevant, this review will specify whether cells and/or nuclei are used (e.g., sc or sn), particularly in technical sections where this distinction is important.

### 3.2. Methods of Single-Cell Transcriptomics and Their Application in Postmortem Human Brain

While specific methodological details of single-cell transcriptomics depend largely on technology, input material, and research questions, the general steps include isolation of cells/nuclei, purification (as needed) and quality check of cells/nuclei, counting of cells/nuclei, capture of cells/nuclei, lysis of cells/nuclei, reverse transcription of RNA to complementary DNA (cDNA), cDNA amplification, and library preparation for sequencing. Detailed reviews have been published further outlining single-cell transcriptomic methodologies, applications, advantages, and limitations [1,5,6,7,11,13,15,16,27].

#### 3.2.1. Sample Preparation

Common isolation methods of individual cells include flow cytometry or fluorescence-activated cell sorting (FACS), magnetic-activated cell sorting (MACS), laser capture microdissection (LCM), manual cell picking, random seeding/dilution, and microfluidic devices [11,64,99,100]. Most single-cell transcriptomic studies in frozen postmortem human brain use nuclei rather than cells for reasons described above [91]. Nuclei isolation is typically performed by lysing cells through chemical (e.g., low-concentration detergent) and physical (e.g., dounce homogenization) dissociation of tissue samples often prepared by cryosection or macro-dissection with a scalpel [73,75,76,77,78,79,80,81,101].

The resulting nuclei suspension is generally filtered through cell strainers and washed with a PBS-based buffer, followed by a form of purification to reduce cellular debris and ambient RNA in the suspension [72,73,75,76,77,78,79,80,81,82,101]. Many methods have been developed for nuclei purification, and most include a density gradient, ultracentrifugation, or FANS for purification [72,73,75,77,79,81,82]. There are commercially available kits for nuclei isolation and purification as well, some of which have already been used successfully with human brain tissue, while others show promise for implementation based on results from human cell cultures and frozen brain tissue of other species (e.g., mouse): Millipore Sigma Nuclei EZ Prep (Sigma-Aldrich, St. Louis, MO, USA), 10x Genomics Chromium Nuclei Isolation Kit (10x Genomics, Pleasanton, CA, USA), Miltenyi Anti-Nucleus MicroBeads (Bergisch Gladbach, Germany), Miltenyi Debris Removal Solution (Bergisch Gladbach, Germany), and Miltenyi Myelin Removal Beads II (Bergisch Gladbach, Germany) [102,103,104]. Researchers have also modified protocols and microfluidic devices for nuclei isolation and capture [76,77,103]. While modified microfluidic devices, commercial kits, and FANS can be prohibitively costly for researchers, other alternatives such as sucrose and iodixanol density gradients (e.g., Millipore Sigma Optiprep density gradient medium, Sigma-Aldrich, Saint louis, MO, USA) offer more cost-effective approaches for nuclei purification [76,80,88,101]. Brain tissue is particularly difficult to generate purified, single nucleus isolations from, as it is abundant in lipid-rich myelin that is difficult to remove and can interfere with downstream analysis [23,105]. More specific to frozen postmortem human brain tissue is that there is a trade-off between nuclei purity, integrity, quantity, and RNA integrity. For example, FANS exerts pressure on nuclei, which can lead to damage and loss of nuclei and RNA. However, FANS provides a well-purified suspension of isolated nuclei.

For quality checks, cells are often stained with acridine orange/propidium iodide (AO/PI) or Trypan Blue to determine viability by fluorescent microscope or automated cell counter [23]. Quality checks for nuclei often include staining with Hoechst or DAPI (stains DNA) and wheat germ agglutinin (WGA) (binds nuclear pores to stain the nuclear membrane) to access quality under a fluorescent microscope—good quality nuclei with an intact nuclear membrane should be stained with both [23,106]. Counting of both cells and nuclei is often performed by an automated cell counter, hemocytometer, or flow cytometer [23,75,77,78,80,81,82].

Experimental variability can result from variation in these sample preparation steps, and community standards are currently lacking. Key directions in standardizing and optimizing sample preparation, particularly for postmortem human brain, include collaborative efforts between researchers to develop standards for efficient sample processing and consensus on quality checks and control measures [23].

#### 3.2.2. Single-Cell Capture

Cells/nuclei, along with their RNA content, can be individually captured by plate or droplet-based assays. Plate-based methods isolate individual cells/nuclei or groups into wells using FACS/FANS [75,107] or patch pipette capillary [108]. Cells/nuclei are subsequently lysed, RNA reverse transcribed then amplified. Each well is either processed separately or pooled for sequencing. Advantages of these methods are that they provide sensitive gene detection and are customizable, but they are often high cost per cell, low cellular and molecular throughput, and may be subject to operator bias for cell sorting [21,23,27,109]. Droplet-based methods simultaneously analyze thousands of individual cells/nuclei encapsulated alongside a barcoded microparticle in oil droplets created by a microfluidic device. Individual cells/nuclei are subsequently lysed and RNA content bound to oligonucleotides on barcoded beads that contain distinct primer sequences, generally with additional cell barcodes, PCR handles, and UMI sequences. The RNA is then reverse transcribed into cDNA and amplified to generate libraries for sequencing. Advantages of these methods include efficiency, low cost per cell, and high cellular and molecular throughput, but they require specialized microfluidic equipment and pose challenges with bead–cell encapsulation, resulting in more than one cell/nucleus or empty droplets [21,23,27,109]. Choice of single-cell capture method depends largely on technology sensitivity and efficiency of RNA capture and conversion to cDNA, accuracy and precision of RNA quantification, technical complexity, and cost [23,27].

Newer methods to obtain single-cell transcriptomic information also exist that do not require the isolation of individual cells/nuclei for capture, including technologies that use combinatorial barcoding/indexing strategies, such as SPLiT-seq [110], sci-RNA-seq [111,112], and sci-L3 [113]. Combinatorial barcoding/indexing approaches tag nucleic acid contents of cells/nuclei by adding unique cell-specific oligonucleotides called barcodes/indexes [110,111,112,113]. Cells/nuclei are distributed in wells for contents to be tagged with barcodes/indexes, then pooled and split into wells again to be tagged with different barcodes/indexes. The process is repeated for molecules to acquire random barcode/index combinations that allow separation and assignment to respective cells computationally. These approaches are scalable and low cost per cell, but they are often limited by laborious workflows requiring many rounds of pipetting, and they are not well suited for lower cellular input [27]. A more recent single-cell transcriptomic method that also does not require the isolation of individual cells/nuclei for capture is particle-templated instant partition sequencing (PIP-seq) [114]. PIP-seq creates uniform droplet emulsions, each containing an individual cell/nucleus and a barcoded bead by vortexing thousands to millions of cells/nuclei [114]. Compared to combinatorial barcoding/indexing strategies, PIP-seq has a simpler workflow, intrinsic scalability (10 to over 10^6^ cells), and allows genome-wide high-throughput processing, which can profile many samples in parallel [114].

For a more thorough review of these single-cell capture methods, see Sant et al. [21], Shang et al. [22], Regan et al. [23], and Ziegenhain et al. [27].

#### 3.2.3. Library Construction

An important difference between these single-cell capture technologies is how they capture and process RNA. Two general methods exist for producing single-cell sequencing libraries from captured RNA: tag-based or full-length approaches. Droplet- and plate-based methods are often tag-based or full-length, respectively. These methods can both generate cDNA libraries for sequencing.

The tag-based approach captures either the 3′ or 5′ end of polyadenylated RNA, mainly mRNA, but other polyadenylated RNA (e.g., long non-coding RNA and primary microRNA) may be captured. Both the 3′ and 5′ methods capture RNA that becomes cDNA with a unique cell barcode (i.e., identifies cell of origin) and UMI (i.e., unique to transcript for PCR bias correction). Methods target the 3′ end by using poly(dT) terminal sequences on the bead oligonucleotides, which bind the 3′ end of polyadenylated RNAs, while methods that target the 5′ end use a template-switching oligo (TSO) containing poly(G) as the terminal sequences of the bead oligonucleotides and poly(dT) as reverse transcription primers to target polyadenylated RNA. A transcriptase adds poly(C) to the 3′ end of the synthesized cDNA, which binds the poly(G) TSOs. The template switching occurs when the transcriptase switches template from the poly(C) target sequence to the TSO [115,116]. This provides cDNA with known sequences at both the 3′ and 5′ ends to be efficiently amplified and easily identified. This template switching mechanism is commonly used and is also present in the 3′ end method where the TSO is added to the sample suspension before capture [117]. 3′-based methods are most commonly used, but 5′-based methods are best for immune profiling of cells such as B and T cells [21,22,23,27]. 3′ tag-based methods are best for large-scale profiling focused on gene identity and expression levels, as they offer high-throughput capabilities with UMIs for accurate transcript identification, but they are limited in isoform detection [27]. Tag-based methods include Chromium 3′ and 5′ Gene Expression [117], snDrop-seq [77], Drop-seq [70], InDrop/Drops [69,118], CEL-seq/seq2 [119,120], STRT-seq [121], MARS-seq/seq2.0 [74,122], DroNc-seq [103], PIP-seq [114], and Seq-Well [123].

The full-length approach profiles entire RNA transcripts, most often polyadenylated. In general, poly(dT) primers and template switching are used to generate cDNA. Later iterations of full-length methods such as Smart-seq3 include UMIs for improved accuracy in transcript quantification [124]. Additionally, it is possible to profile a broad spectrum of RNA that are not polyadenylated with methods such as Smart-seq-total [125]. Full-length methods profile whole RNA transcripts, thereby reducing 3′ or 5′-end bias, producing uniform and representative cDNA libraries, and allowing for more detailed detection of transcriptomic features. These techniques are therefore best for investigating gene regulation, gene isoforms, rare transcripts, and alternative splicing events but are lower cellular and molecular throughput and often more labour-intensive for numerous samples than tag-based approaches [27]. Larger sample sizes and high throughput are often needed for robust analysis of differentially expressed genes in neuropsychiatric disorders due to their high heterogeneity [6]. Full-length methods include Smart-seq/seq2/seq3/seq3xpress [124,126,127,128], MATQ-seq [129], and FLASH-seq [130].

Advancements in single-cell transcriptomic technologies have produced tag-based and full-length library construction that is compatible with both plate- and droplet-based platforms. For example, VASA-seq profiles polyadenylated RNA and non-polyadenylated RNA [131]. The combination of these library construction methods in VASA-seq increases the amount of information that can be uncovered, such as alternative splice variants [131].

#### 3.2.4. Single-Cell Transcriptomic Technologies Applied to Postmortem Human Brain

Tag-based snRNA-seq methods are often used to research neuropsychiatric disorders in the human brain, since larger sample sizes and high cellular and molecular throughput are needed to detect significant gene expression changes [6]. Specifically, droplet- and 3′ tag-based microfluidic methods are currently popular for snRNA-seq in postmortem human brain due to their robustness, low cost per cell, high throughput, commercial availability, and relative ease of use [36,37,38,42]. These include Chromium 3′ Gene Expression [45,72,73,78,80,81,87,88,132,133,134,135,136,137,138,139,140,141,142], snDrop-seq [77], and DroNc-seq [103] (Table 2). Smart-seq/seq2, which now has newer versions, is a full-length, plate-based method that has also been applied to postmortem human brain [75,86] (Table 2).

One of the first studies to demonstrate the feasibility and validity of using frozen postmortem human brain tissue for snRNA-seq used FANS to sort individual NeuN+ nuclei from the prefrontal cortex into a microplate for Smart-seq2 [75]. Another study also used FANS to sort NeuN+ nuclei from six cortical regions for assay through Fluidigm C1 Single-Cell Auto Prep Array for mRNA-seq [76]. Soon after, DroNc-seq was created and applied to frozen postmortem human prefrontal cortex and hippocampus samples, thus demonstrating the ability to apply droplet-based high-throughput snRNA-seq to nuclei from frozen postmortem human brain [103]. Similarly, snDrop-seq was developed and applied to postmortem adult human visual cortex, frontal cortex, and cerebellum [77]. Both DroNc-seq and snDrop-seq incorporated adjustments to Drop-seq for optimized use of nuclei, such as efficient processing of the nuclei and alterations of the microfluidic device dimensions [77,103].

While not yet published in postmortem human brain research, some newer methods show great promise for application. For example, Chromium Flex Gene Expression, 10x Genomics, California, USA uses probe-based chemistry for whole-transcriptome profiling and allows the fixation of nuclei, which can be convenient for multiplexing samples, optimizing sample preparation workflow, minimizing technical batch effects, and using lower quality tissue such as archival samples. Additionally, methods like snPATHO-seq [143] and snRandom-seq [144] are being developed to use more types of preserved tissue for single-cell transcriptomics, such as FFPE tissue.

Single-cell transcriptomics has facilitated ground-breaking developments in human brain research, providing various insights into the biology of many neuropsychiatric disorders, including Alzheimer’s disease, autism spectrum disorder, major depressive disorder, post-traumatic stress disorder, schizophrenia, and many others [72,73,78,80,81,82,84,85,87,88,132,133,134,137,138,141,145] (Table 2). The largest current single-cell transcriptomic dataset for the human brain, generated using the Chromium 3′ Gene Expression platform, consists of 6.3 million nuclei from the dlPFC of 1494 donors [138]. This cohort represents 20 neurodegenerative/neurological diseases and 13 mental health disorders, ranging from more prevalent (Alzheimer’s disease, *n* = 519; schizophrenia, *n* = 177) to less prevalent (obsessive–compulsive disorder, *n* = 6; post-traumatic stress disorder, *n* = 2) [138]. The authors shared this dataset as an online repository to foster collaborative research across the scientific community [138]. A significant benefit of single-cell transcriptomic studies is that the large amount of generated data is often made publicly available, allowing reuse by other researchers. The reuse of single-cell transcriptomic data can often result in additional findings to the original study, as computational analyses can differ and improve, enabling deeper exploration of these datasets.

**Table 2:** Single-cell omic technologies applied in postmortem human brain.

	Modality									
		Epigenomics								
Method	Transcriptomics	DNA Methylation	Histone Modifications	3D Genomic Structure	Chromatin Accessibility	Single-Cell Capture Strategy	Tissue Type Used in Cited Studies	Technique Publication	Applied in Postmortem Human Brain	Applied in Neuropsychiatric Disorders
snDrop-seq	✓					Droplet-based(MF)	FF-nuclei	[77]	[77]	
DroNc-seq	✓					Droplet-based(MF)	FF-nuclei	[103]	[103]	AD
Chromium 3′ Gene Expression	✓					Droplet-based (MF)	FF-nuclei	[117]	[45,72,73,78,79,80,81,87,88,132,133,134,135,137,138,139,140,141,145]	MDD, PTSD, OCD, SCZ, SCA, AD, ASD, AN, BN, PD, BD, PTSD, FTD, ADHD, AUD
SnISOr-seq/seq2	✓					Droplet-based (MF)	FF-nuclei	[146,147]	[146,147,148]	FTD
Smart-seq/seq2	✓					Plate-based (SW)	FF-nuclei	[127,128]	[75,86]	
sciMETv2/v3		✓				Plate-based (CI)	FF-nuclei	[149,150]	[149,150]	
Drop-BS		✓				Droplet-based (MF)	FF-nuclei	[151]	[151]	
sciEM		✓				Plate-based (CI)	FF-nuclei	[152]	[152]	
snmC-seq/-seq2/-seq3		✓				Plate-based (SW)	FF- nuclei	[153,154,155]	[153,154,156]	
sciMET-CAP		✓				Plate-based (CI)	FF-nuclei	[157]	[157]	
nano-CUT&Tag *			✓			Droplet-based (MF)	FF-nuclei	[158]	[159]	
Chromium Epi ATAC					✓	Droplet-based (MF)	FF-nuclei	[160]	[132,134,137,141,161,162,163,164,165]	MDD, AD, SCZ, SCA, PD, BD
txci-ATAC-seq					✓	Plate-based (CI) & Droplet-based (MF)	FF-nuclei	[166]	[166]	
scTHS-seq					✓	Plate-based (CI)	FF-nuclei	[77]	[77]	
Chromium Epi Multiome ATAC + Gene Expression	✓				✓	Droplet-based (MF)	FF-nuclei		[132,137,161,167,168]	MDD, PTSD, PD, AD, AUD
SnISOr-ATAC	✓				✓	Droplet-based (MF)	FF-nuclei	[169]	[169]	AD
MUSIC	✓			✓		Plate-based (CI) & Droplet-based (MF)	FF-nuclei	[170]	[170]	AD
snm3C-seq/-seq3		✓		✓		Plate-based (SW)	FF-nuclei	[171,172]	[156,171,172]	
snmCAT-seq/snmCT-seq	✓	✓			✓	Plate-based (SW)	FF-nuclei	[173]	[173,174]	

* Only available as a preprint. CI = combinatorial indexing; MF = microfluidics, SW = single well. FF = fresh-frozen; AD = Alzheimer’s disease; PD = Parkinson’s disease; MDD = major depressive disorder; BD = bipolar disorder; SCZ = schizophrenia; SCA = schizoaffective disorder; PTSD = post-traumatic stress disorder; ASD = autism spectrum disorder; OCD = obsessive–compulsive disorder; AUD = alcohol use disorder; FTD = frontotemporal dementia; AN = anorexia nervosa; BN = bulimia nervosa; ADHD = attention deficit hyperactivity disorder.

## 4. Spatial Transcriptomics

The rise of single-cell omics led to the development of *spatially resolved* transcriptomics, which was awarded Nature’s method of the year in 2020 [175,176]. Spatial transcriptomics localizes, profiles, and visualizes RNA in single, whole cells to evaluate spatial patterns of gene expression and cellular organization. Attaining spatial context for complex tissues, such as the brain, is of particular importance, as the brain is heterogeneous in structure, regional specialization, and cellular composition [45,177,178]. As such, it is imperative to acquire spatial information from brain cells to inform cell identity, connectivity, and function, thus revealing cellular networks that shed light on overall brain function. Spatial transcriptomic methods have advanced by improving precision, resolution, tissue coverage, and multiplexing capabilities [19,179]. These advances have led to spatial transcriptomic technologies that have subcellular resolution, are increasingly commercially available and user-friendly, and have been successfully implemented in postmortem human brain tissue (Table 3) [49,72,139,140,180,181,182,183]. These pivotal technological developments and direct applications to the brain have resulted in many recent novel findings and substantial contributions to the existing knowledge of the organ. Key contributions from spatial transcriptomics include the elucidation of spatiotemporal molecular dynamics of the developing human brain, insights into adult brain plasticity, cell type and area specificities of brain regions, species-specific features in organization of the human brain, and cell type and state differences in neuropsychiatric disorders [49,83,136,139,140,180,181,182,183,184,185].

### 4.1. Methods of Spatial Transcriptomics and Their Application in Postmortem Human Brain

Spatial transcriptomic methods differ in fundamental technological properties and therefore obtain different information from each other (e.g., spatial resolution and coverage of transcriptome). Specific methodological details and the information to be obtained depend largely on technology, input material, and research questions—similarly to single-cell transcriptomics. Different spatial transcriptomic methods can be applied to FF, FxF, or FFPE human brain tissue (Table 3). Current technologies can be broadly categorized based on how they obtain spatial information, either through imaging- or sequencing-based methods. These methods all pose advantages and limitations that are a trade-off between resolution, tissue scalability, cost, gene detection capabilities, and overall efficiency [19,29]. There have been detailed reviews published about these methods and their applications, advantages, limitations, and computational methods [1,5,6,10,13,14,18,19,20,24,25,29].

#### 4.1.1. Imaging-Based Spatial Transcriptomics

Early efforts of spatially mapping gene expression included in situ hybridization (ISH), which used autoradiography to detect a radioactively labeled RNA or DNA probe hybridized to a target gene in tissue [186]. A key development to this technology resulted in fluorescence in situ hybridization (FISH), where the radioactive probes were replaced with fluorophore-conjugated oligonucleotides that can be visualized using fluorescent microscopy [187]. This led to improved resolution with the introduction of single molecule fluorescence in situ hybridization (smFISH), which uses super-resolution microscopy to visualize fluorescent probes that bind individual RNA molecules [188]. The focus for technological development of imaging-based spatial transcriptomic methods over the past years has been on improving multiplexing capacity, signal intensity, and gene detection range [10,19,29]. These improvements have been made by improving probe chemistry, error-robust barcoding, and advancing high-resolution optical imaging, thus greatly expanding the multiplexing capacity of FISH-based assays. Image-based methods also incorporate in situ sequencing (ISS) as an alternative approach to FISH, allowing nucleotide sequences to be read directly from tissues [189].

Imaging-based spatial transcriptomics uses multiplexed ISH or ISS with error-correcting barcodes combined with high-resolution fluorescence microscopy to visualize, discriminate, and quantify fluorescent signals from probes bound to RNA molecules in their native location within tissue sections, thus enabling precise mapping of gene expression, often to subcellular resolution. Gene-specific fluorescent probes undergo a distinct number of hybridization rounds, each encoding a barcode that identifies the target gene. Current imaging-based spatial transcriptomic methods include seqFISH/FISH+ [190,191], MERFISH/MERSCOPE [192], RNA SPOTs [193], osmFISH [194], FISHnCHIPs [195], STARmap [196], EEL FISH [197], HybISS [198], and Xenium [199]. Imaging-based spatial transcriptomic technologies can provide very high spatial resolution, precision, and tissue coverage but are often limited by molecular throughput, optical crowding, targeted approaches, cost, and technical complexity [10,19,29].

#### 4.1.2. Sequencing-Based Spatial Transcriptomics

The development and enhancement of techniques such as laser capture microdissection (LCM) [200] and RNAscope [201] were pivotal in the development of sequencing-based spatial transcriptomics. Early adaptations of these methods targeted regions of interest by combining LCM with single-cell transcriptomic techniques such as LCM-Seq [202] and GEO-seq [203]. Another early targeted approach was tomo-seq [204]. The latest sequencing-based spatial transcriptomic approaches permit unbiased, transcriptome-wide analysis while retaining spatial coordinates over large, biologically relevant tissue areas [10,19,29]. The location of transcripts can be revealed by combining spatial barcoding strategies with sequencing. These methods have rapidly gained popularity for large-scale studies, as they provide greater molecular throughout and coverage compared to imaging-based spatial transcriptomic methods [10,19,29].

Sequencing-based spatial transcriptomic methods localize gene expression in tissue sections through spatially indexed capture of RNA by unique oligonucleotide barcodes on surfaces (e.g., slide, microarray, bead, and microfluidic channels), each corresponding to a discrete location. This is followed by library preparation and sequencing. Current sequencing-based spatial transcriptomic technologies include Visium/VisiumHD [175,205], DBiT-seq/xDBiT [206,207], Slide-seq/seqV2 [208,209], Stereo-seq/V2 [210,211], GeoMx Digital Spatial Profiler (GeoMx DSP) [212], Seq-Scope [213], HDST [214], and Pixel-seq [215]. Sequencing-based spatial transcriptomic approaches provide very high molecular throughput, unbiased RNA detection at the whole-transcriptome level, and ease of use and reproducibility with increased commercial availability but are limited by spatial resolution (10–55 µm for most, some less than 2 µm), sequencing data that correspond to a spot—not necessarily a single cell, cost, and detection efficiency of RNA [10,19,29].

#### 4.1.3. Spatial Transcriptomic Technologies Applied to Postmortem Human Brain

As previously discussed, brain cells are highly interconnected, and dissociation of brain tissue can damage cells and alter transcription profiles [91,92]. The development of spatial transcriptomics has been exceptionally beneficial for the study of the brain, as the isolation of cells or nuclei is not necessary. Additionally, the use of whole tissue sections allows for the profiling of cytoplasmic RNA and cellular subcompartments in postmortem human brain. Methods that have been used for spatial transcriptomics in human postmortem brain tissue include Visium [49,72,139,140,180,183], Xenium [132], GeoMx DSP [141,216,217], EEL FISH [197], HybISS, Stereo-seq [218], and MERFISH/MERSCOPE [142,185] (Table 3).

Neuropsychiatric disorders that have been investigated by spatial transcriptomics in postmortem human brain tissue include schizophrenia, Parkinson’s disease, Alzheimer’s disease, autism spectrum disorder, post-traumatic stress disorder, and major depressive disorder [49,72,132,139,163,180] (Table 3). While these experiments are limited by sample size, with only one or few donors, they showcase how spatial transcriptomics can advance our understanding of neuropsychiatric disorders.

One study that was among the first to apply spatial transcriptomics to postmortem human brain used Visium in the dlPFC of three adult males and integrated their data with clinical gene sets for multiple neuropsychiatric disorders (e.g., psychENCODE) [49,84]. The study found layer-enriched expression of genes linked to schizophrenia and, to a lesser extent, bipolar disorder in L2 and L5 [49]. Additionally, heritability partitioning analysis found heritability is enriched for L2-enriched genes for schizophrenia and bipolar disorder [49,219]. Maynard et al. [49] also overlaid Chromium 3′ Gene Expression data in two donors and other publicly available snRNA-seq data to validate these laminar expression signatures and to enhance the annotation of gene expression-driven clusters [49]. These findings underscore the need for spatially defined gene expression patterns to gain more information about neuropsychiatric disorders. In addition to these scientific contributions, the group created a web application, spatialLIBD, to access, visualize, and explore raw and processed spatial gene expression data [49].

**Table 3:** Spatial omic technologies applied in postmortem human brain.

	Modality									
		Epigenomics								
Method	Transcriptomics	Chromatin Accessibility	Proteomics	Metabolomics	Tissue Requirements	Resolution	Imaging-Based or Sequencing-Based	Technique Publication	Applied in Postmortem Human Brain	Applied in Neuropsychiatric Disorders
MERFISH/MERSCOPE	✓				Type: FF; FxF; FFPE Size: 2 × 1.5 cm Thickness: 10 µm	Subcellular	Imaging	[192]	[142,185]	AD
EEL FISH	✓				Type: FF Size: 24 × 60 mm Thickness: 10 µm	Single-cell	Imaging	[197]	[197]	
HybISS	✓				Type: FF Size: 25 × 75mm Thickness: 5–20 µm	Subcellular	Imaging	[198]	[198]	
Xenium	✓				Type: FF; FFPE Size: 10.45 × 22.45 mm Thickness: 10 µm-FF; 5 µm-FFPE	Subcellular	Imaging	[199]	[132]	PTSD, MDD
Visium	✓				Type: FF; FxF; FFPE Size: 6.5 × 6.5 mm Thickness: 5–35 µm	55 µm	Sequencing	[175]	[49,72,139,140,180,183]	SCZ, AD
Stereo-seq	✓				Type: FF, FxF, FFPE Size: 13.2 × 13.2 cm Thickness: 5–10 µm	0.22 µm	Sequencing	[210]	[218]	AD
Spatial-ATAC-seq		✓			Type: FF; FFPE Size: 5.5 × 5.5 mm Thickness: 7–10 µm	20 µm	Sequencing	[220]	[220]	
GeoMx DSP	✓		✓		Type: FF; FFPE Size: 35.3 × 14.1 mm Thickness: 5 µm	10 µm	Sequencing	[212]	[141,216,217]	ASD, PD, AD
SMA	✓		✓	✓	Type: FF Size: 5.5 × 5.5 mm Thickness: 10–12 µm	55 µm	Sequencing	[221]	[221]	PD

FF = fresh-frozen; FxF = fixed frozen; FFPE = formalin-fixed, paraffin-embedded. AD = Alzheimer’s disease; PD = Parkinson’s disease; MDD = major depressive disorder; SCZ = schizophrenia; PTSD = post-traumatic stress disorder; ASD = autism spectrum disorder.

## 5. Single-Cell Epigenomics

The study of cell-specific gene expression patterns is essential to understanding the human brain and neuropsychiatric disorders; however, this is only one piece of a complex biological story. Single-cell epigenomics augments the molecular narrative of this story by allowing the investigation of regulatory mechanisms that govern gene expression within individual cells. The study of these regulatory epigenetic mechanisms, including chemical modifications of DNA and histones and chromatin conformational changes, is therefore crucial to understanding the molecular landscape of neuropsychiatric disorders. Each epigenetic mechanism differently influences gene regulation.

The application of NGS, cell and molecular barcoding/indexing, and enhanced isolation techniques in single-cell transcriptomics paved the way for the development of single-cell epigenomics [111,222,223,224,225]. Current single-cell epigenomic methods apply different modalities of epigenetic exploration to individual cells to investigate regulatory programs directing cell identity and function. These modalities provide the capability to examine the molecular dynamics of DNA, histones, and chromatin in exceptional detail [77,149,155,156,226,227,228,229,230,231,232,233]. Recent methodological advancements in single-cell epigenomics include increasing the throughput of cells by introducing combinatorial barcoding/indexing in place of single-well reactions and the development of targeted approaches to reduce sequencing burden [157,227,234]. Similarly to snRNA-seq, single-cell epigenomic assays benefit from their application to frozen postmortem human brain tissue by taking advantage of the robustness of nuclei compared to cells [77,91,162]. Despite a slower adaptation than single-cell transcriptomics, technological progression of single-cell epigenomics has rapidly resulted in many significant findings in the human brain. Most notably, cell type and brain region-specific chromatin accessibility patterns in the developing brain leading to cell type diversity and cell fate determination, uncovering of cell type-specific regulatory elements and transcription factors in different brain regions, and elucidating cell type-specific regulatory mechanisms in neuropsychiatric disorders [77,162,165,235].

### 5.1. Methods of Single-Cell Epigenomics and Their Application in Postmortem Human Brain

Single-cell epigenomic methods provide the ability to examine DNA, histones, and chromatin through diverse methods at the single-cell level. This includes single-cell analysis of DNA methylation, histone modifications, 3D genomic structure, and chromatin accessibility to reveal regulatory programs underlying cell identity and function [77,149,155,156,226,227,228,229,230,231,232,233].

#### 5.1.1. DNA Methylation

Most DNA methylation techniques involve chemical or enzymatic conversion of unmethylated cytosines (C), as conventional sequencing technologies do not distinguish between methylated and unmethylated cytosines [236]. The most common method to assess DNA methylation is bisulfite conversion, where unmethylated cytosines are converted to uracil (U), while methylated cytosines are not [236]. However, bisulfite conversion has limitations: long reaction times, potential DNA damage, erroneous conversion (e.g., failure to convert unmethylated cytosines or inappropriate conversion of 5-methylcytosine (5mC) to thymine), and failure to distinguish between 5mC and its oxidized derivative, 5-hydroxymethylcytosine (5hmC), which has been proposed to play a distinct physiological role [237,238,239,240]. In contrast, enzymatic conversion methods use three separate enzymes to first protect 5mC and 5hmC from deamination, then to convert unmethylated cytosines to uracil, resulting in reduced DNA damage and improved GC distribution [241]. At the single-cell level, the majority of techniques continue to rely on bisulfite conversion [149,151,155,242], although methods utilizing enzymatic conversion have recently emerged, as well as a targeted probe-based approach using a DNAm-sensitive endonuclease [150,152,234]. Moreover, post-bisulfite adaptor tagging (PBAT), in which sequencing adapters are ligated after bisulfite conversion, has been applied to circumvent the loss of intact sequencing templates caused by bisulfite-induced DNA damage [243,244]. Furthermore, methods with the capacity to distinguish 5mC from 5hmC have been developed [245,246].

Single-cell DNA methylation techniques can be further categorized based on whether they utilize single-well reactions (e.g., snmC-seq3, scBS-seq, and scWGBS); combinatorial barcoding/indexing (e.g., sciMET/v2/v3); or microfluidic systems (e.g., scTAM-seq and Drop-BS) [149,151,155,156,234,242,247]. Single-well reactions typically involve sorting purified single cells/nuclei into plates, converting unmethylated cytosines via bisulfite or enzymatic methods, PCR, and sequencing [153,154,155,156,171,233]. Due to high processing times, single-well techniques typically have low cellular throughput unless plate processing can be automated by robotics, such as in snmC-seq3 [24,155]. To improve cellular throughput and processing times, several combinatorial barcoding/indexing and microfluidic techniques have been developed. In combinatorial barcoding/indexing methods, nuclei are subject to first-level barcoding/indexing with a Tn5 complex and second-level barcoding/indexing during a later PCR step; as a result, each nucleus possesses a unique combination of tagmented and PCR barcodes/indexes [149,152]. In microfluidic approaches, cells/nuclei are partitioned into droplets alongside barcoded beads to achieve single-cell resolution similarly to microfluidic-based approaches for single-cell transcriptomics [151,234].

#### 5.1.2. Single-Cell DNA Methylation Technologies Applied to Postmortem Human Brain

Single-cell DNA methylation techniques that have been applied to study postmortem human brain include sciEM [152], sciMETv2/v3 [149,150], snmC-seq/seq2/seq3 [153,154,156], and Drop-BS [151] (Table 2). These studies demonstrate that DNA methylation may be examined at single-cell resolution in postmortem human brain tissue using microfluidics (Drop-BS), combinatorial barcoding/indexing (sciEM and sciMETv2/v3), or single-well (snmC-seq/seq2/seq3) techniques (Table 2). Furthermore, while most of these techniques use bisulfite conversion, sciEM demonstrates that enzymatic methyl conversion is also compatible with postmortem human brain [152]. In addition to demonstrating that DNA methylation signatures can be used to identify distinct cell types in human brain, these studies have also highlighted species-specific differences in DNA methylation, as well as differences between brain regions [151,153,156]. In particular, Tian et al. [156] identified 40 major cell types and 188 subtypes across 46 brain regions from three adult males based on their combined CG and CH methylation signatures, serving as an atlas of DNA methylation in postmortem human brain. They also introduced single-cell methylation barcodes (scMCodes) to aid in the identification of human brain cell types [156]. However, as the aforementioned studies primarily served as proofs of concept for the techniques described, they were limited by their low sample sizes (*n* = 1–4) [149,150,151,152,153,154,156].

One major limitation in single-cell DNA methylation studies is the high sequencing burden required to detect enough methylated sites to perform cell type assignment [157]. A recent paper by Acharya and colleagues [157] expanded on their previously published combinatorial barcoding/indexing method, sciMETv2, by leveraging a methylome capture panel to enrich for known regulatory regions, thereby reducing reads required to achieve comparable cell type identification [149,157]. A more recent paper by the same group further expanded on the sciMET protocol by adapting the method for use with enzymatic conversion, as well as bisulfite conversion, and alternate sequencing instrumentation [150]. As neuropsychiatric disorders display considerable heterogeneity [178], assessing DNA methylation differences at the population scale requires a sizable cohort. The advances to the sciMET protocol, particularly the increase in cellular throughput by combinatorial barcoding/indexing and the reduction in sequencing burden enabled by the compatibility with target enrichment panels, increase the accessibility of population-scale studies, therefore showing great promise for the application to postmortem human brain tissue for the study of neuropsychiatric disorders.

#### 5.1.3. Histone Modifications

The most well-known method for profiling histone modifications is chromatin immunoprecipitation followed by sequencing (ChIP-seq) [248,249]. In this technique, histone proteins are crosslinked to DNA. Target-specific antibodies are used to isolate the histone modification of interest, and sequencing libraries can be generated. The adaptations of ChIP for single cells (e.g., Drop-ChiP and scChiP-seq) use microfluidic devices to encapsulate individual cells in droplets where a micrococcal nuclease (MNase) digests exposed chromatin and makes nucleosomes accessible, while unique barcoded adapters are used to label the chromatin of each cell, conferring single-cell analysis [231,250,251]. Cells are then subjected to ChIP-seq to obtain information about cell-specific DNA–protein interactions, such as histone modifications [231,250].

As an alternative to ChIP, chromatin immunocleavage (ChIC) was developed as an approach that leverages target-specific antibodies to cleave DNA at specific protein–DNA binding sites [252]. In ChIC, an antibody enters intact cells/nuclei and binds to the protein/modification of interest (e.g., H3K27me3). Then, a fusion product of Protein A and MNase (pA-MN) binds to the antibody and cuts the DNA surrounding it. A modification of ChIC, CUT&RUN, is adapted for deep-sequencing applications [232]. Methods based on both ChIC and CUT&RUN have been applied at the single-cell level to profile histone modifications (e.g., uliCUT&RUN, scChiC-seq, and iscChIC-seq), with varying cell throughputs [253,254,255].

To improve the compatibility of these methods with high-throughput single-cell platforms, Kaya-Okur and colleagues [256] developed CUT&Tag. This method uses a Tn5 transposase containing sequencing adapters instead of MNase, resulting in PCR ready DNA fragments. Unlike with CUT&RUN, the transposed DNA fragments in CUT&Tag remain in the nucleus and are thus compatible with microfluidic and combinatorial barcoding/indexing systems post-tagmentation [226,256,257,258]. Additionally, simultaneous mapping of multiple histone marks and joint profiling of histone modifications and transcriptomes in single cells have been achieved via modifications of the original CUT&Tag protocol (e.g., scCUT&Tag, nano-CUT&Tag, ACT-seq, multi-CUT&Tag, scMTR-seq, and droplet paired-tag) [158,226,228,259,260,261,262]. Alternate techniques that also leverage Tn5 to investigate one or multiple histone marks at the single-cell level include ChIL–seq [263], itChIP-seq [264], CoTECH [265], CoBATCH [266], uCoTarget [267], TIP-seq [268], and sciTIP-seq [268].

#### 5.1.4. Single-Cell Histone Modification Technologies Applied to Postmortem Human Brain

At the time of this review, none of the single-cell methods for investigating histone modifications have been applied in postmortem human brain in a peer-reviewed journal. However, one study using nano-CUT&Tag has been published as a preprint, demonstrating that exploring single-cell histone modifications in postmortem human brain tissue is a promising avenue for research [159] (Table 2). Additionally, the success of several of these protocols applied to either mouse brain or human cell culture suggests that progression of single-cell histone modification studies in postmortem human brain may not be far behind [158,226,259,267].

#### 5.1.5. 3D Genomic Structure

Investigations of 3D genomic structure have conventionally involved the use of formaldehyde to create covalent crosslinks between strands of DNA that are physically close regardless of their proximity along the linear sequence of the genome [269]. In the standard Hi-C method, a cocktail of restriction enzymes is used to digest DNA, leaving behind single-stranded ends that are filled with biotin-labeled nucleotides. Nearby fragments are ligated together, leading to the formation of chimeric fragments with biotin at the junction [269]. In single-cell applications of Hi-C, the fixation through the ligation steps are performed in bulk suspensions, then subsequent steps are performed on individually sorted nuclei. In brief, crosslinks are reversed, DNA is fragmented, streptavidin-coated magnetic beads are used to pull down the chimeric fragments, and captured fragments are indexed for sequencing [230,270,271]. Adaptations of the single-cell Hi-C protocol include omitting biotin pulldown to minimize DNA loss and leveraging high-coverage whole-genome amplification with multiplexed end-tagging amplification (e.g., Dip-C and snm3C-seq) [156,171,172,272,273]. The cellular throughput of single-cell 3D genomic methods has been improved by the use of microfluidics and combinatorial barcoding/indexing approaches. For example, in Droplet Hi-C, post-ligation nuclei are processed through the Chromium Epi ATAC kit for barcoding, whereas, in sciHi-C, barcoded adapters are leveraged for multiple rounds of barcoding [274,275,276]. A limitation of many current 3D genomic methods is the use of restriction enzymes for DNA digestion. Since restriction enzymes possess specific target sequences, the pattern of restriction sites can result in nonuniform genomic coverage [277]. A novel technique using Tn5 transposase to fragment the DNA reduces the bias resulting from restriction enzyme specificity [278]. Although this technology has not yet been applied in human brain or at the single-cell level, it represents a promising avenue for future single-cell 3D genomic methods [278].

#### 5.1.6. Single-Cell 3D Genomic Structure Technologies Applied to Postmortem Human Brain

The 3D genomic structure has been investigated at the single-cell level in postmortem human brain tissue using the snm3C-seq method, which simultaneously profiles DNA methylation [156,171,172] (Table 2). Indeed, all currently available methods for profiling the 3D genome in postmortem human brain are multi-omic in nature [156,170,171,172]. In addition to demonstrating the feasibility of assessing 3D genome structure in postmortem human brain, these studies identified cell type-specific enrichment of short-/long-range chromatin interactions and brain regional differences in chromatin organization [156,171,172]. However, the snm3C-seq method involves single-well reactions, making it a lower cellular throughput technique in the absence of specialized robotic equipment for automating pipetting, thus limiting the accessibility of this method. Proof-of-concept studies demonstrating the use of higher cellular throughput and less expensive techniques in postmortem human brain are necessary to advance our knowledge of higher order chromatin structure in the human brain, particularly for the study of neuropsychiatric disorders. Progress towards this goal has been made with MUSIC, which uses a combined strategy of split pooling and microfluidics to simultaneously profile chromatin interactions and the transcriptome in 14 human frontal cortex samples of the aging brain, including varying stages of Alzheimer’s disease pathology [170] (Table 2).

#### 5.1.7. Chromatin Accessibility

A popular method for assessing chromatin accessibility is the assay for transposase-accessible chromatin with high-throughput sequencing (ATAC-seq). Briefly, isolated cells/nuclei are tagmented using a hyperactive form of the Tn5 transposase, which selectively fragments the accessible ‘open’ regions of chromatin and inserts adapters for sequencing [279]. By integrating their bulk ATAC-seq protocol with a microfluidics platform and leveraging cell barcoding, Buenrostro and colleagues [227] developed a single-cell ATAC-seq method (scATAC-seq). An alternative scATAC-seq/snATAC-seq method was later commercialized, Chromium Epi ATAC, which includes cellular barcoding using gel beads and the microfluidic device previously described [160]. Additional advances in single-cell chromatin accessibility methods include combinatorial barcoding/indexing approaches (e.g., scifi-ATAC-seq, dsciATAC-seq, and txci-ATAC-seq) that leverage a barcoded Tn5 during the transposition step prior to either the microfluidic capture (to surpass the ‘one cell per droplet’ limit) or individual well reactions [165,166,222,280,281,282,283,284].

Alternate methods of investigating chromatin accessibility at the single-cell level include scDNase-seq [229], scMNase-seq [285,286], and scTHS-seq [77]. In scDNase-seq, cells are sorted into individual wells, and the endonuclease DNase 1 is used to digest DNA at hypersensitive sites predominantly within open regions of chromatin, and indexed PCR is used to insert cell barcodes [229,287]. However, this method is limited by cellular throughput due to the single-well reactions. A modified version of scDNase-seq, iscDNase-seq, introduced multiple indexing steps to improve the number of cells processed per experiment [288]. The procedure for scMNase-seq is similar, although it uses a micrococcal nuclease to digest exposed DNA, leading to the recovery of nucleosome-bound DNA [285,286]. The scTHS-seq method introduces linear amplification by IVT alongside a modified Tn5 transposase to increase sensitivity compared to scATAC-seq [77].

#### 5.1.8. Single-Cell Chromatin Accessibility Technologies Applied to Postmortem Human Brain

The existence of the commercial kit, Chromium Epi ATAC, has facilitated the investigation of single-cell epigenomic studies of chromatin accessibility in postmortem human brain using nuclei [132,137,141,162,163,164,165] (Table 2). The findings from these studies show chromatin accessibility differences across brain regions and changes linked to neuropsychiatric disorders in population-scale cohorts, including post-traumatic stress disorder, Alzheimer’s disease, and major depressive disorder [132,162,165]. Furthermore, a study coupled the Chromium Epi ATAC kit with a combinatorial barcoding/indexing approach, txci-ATAC-seq, to improve cellular throughput and demonstrate its compatibility with postmortem human brain tissue [166] (Table 2). Lake et al. [77] used an alternate technique, scTHS-seq, to profile cell type-specific regulatory elements and transcription factors in postmortem human brain (Table 2). These studies generate single-cell resolution chromatin accessibility data from postmortem human brain tissue to elucidate cell-specific epigenetic influences in neuropsychiatric disorders.

### 5.2. Spatial Epigenomics

Spatial epigenomic techniques have recently emerged to provide insight into spatiotemporal epigenetic regulation of gene expression directly in tissues of origin. Epigenomic MERFISH provides *spatially resolved* epigenomic profiles of individual cells by combining CUT&Tag with MERFISH [192,256,289]. Epigenetic modifications of interest can be targeted with an antibody carrying a Tn5 transposase to insert a T7 promotor, producing tagged DNA fragments to be amplified into many RNA copies by in situ transcription [289]. The resulting transcribed RNA is labeled with barcoded FISH probes for multiplexed imaging through MERFISH using sequential rounds of fluorescence microscopy [289]. The development of this technology particularly targeted histone modifications; however, the authors stated that epigenomic MERFISH can be applied to study other epigenetic properties by using appropriate antibodies or other affinity probes [289]. Spatial-CUT&Tag is another newly developed spatial epigenomic method that also uses CUT&Tag [290]. This method enables spatial profiling of histone modifications by combining in-tissue deterministic barcoding [206] with CUT&Tag [256,290]. Antibodies with a Tn5 transposase target histone modifications in tissue, then two rounds of DNA barcoding is performed by microchannel-guided delivery to create a two-dimensional grid of distinctly barcoded tissue pixels [290]. The tissue is then imaged to map spatial epigenomic information to tissue morphology [290]. Finally, reverse crosslinking is performed, and DNA fragments undergo library preparation for NGS [290]. The group also developed a similar method that targets accessible genomic loci rather than histone modifications called spatial-ATAC-seq [220]. While these methods are relatively new and have therefore not been extensively applied, Deng and colleagues applied spatial-ATAC-seq to frozen postmortem human brain tissue (hippocampus and choroid plexus) and identified six differently distributed cell clusters across the tissue that match expected tissue localization [220] (Table 3). This study demonstrated the validity of using spatial-ATAC-seq to spatially resolve brain cell types based on chromatin accessibility profiles. Having already been applied to postmortem human brain, spatial epigenomic technologies show great promise for further application in the study of neuropsychiatric disorders.

## 6. New Horizons in Single-Cell Omics: Multi-Omics and Beyond

The field of single-cell omics is rapidly expanding in various directions. Single-cell multi-omics is of particular interest to study the complexity of the human brain and neuropsychiatric disorders. Singular modality single-cell omics have provided valuable insights to the elaborate molecular biology underlying neuropsychiatric disorders. However, the combination of multiple modalities (i.e., ‘multi’-omics) to simultaneously explore transcriptomic, epigenomic, and other omic information reveals a more complete picture of cells, their identity, states, and interactions. Single-cell multi-omics applies diverse omic techniques that provide varying information, such as dimensionality, allowing for a more comprehensive investigation of the dynamic orchestration of molecular elements that define cellular function. Initial single-cell multi-omic approaches integrated data obtained as separate experimental events [291,292,293], while newer methods can simultaneously profile different omic information in the same cell [8]. These analyses performed jointly in individual cells allow direct elucidation of omic associations in cells. Continuous methodological advancements in single-cell multi-omics include increased capabilities in multiplexing, cell barcoding, throughout, precision, and resolution [8]. In particular, advancements have led to *spatially resolved* multi-omics. Spatial multi-omics provides spatial context for multiple omic modalities simultaneously. Single-cell multi-omic technologies have been applied to postmortem human brain to study different brain regions, the aging brain, and neuropsychiatric disorders [156,168,170,171,172,173].

Other than single-cell multi-omics, future avenues for single-cell omic exploration of the human brain include emerging single-cell technologies to explore more omics, including metabolomics, and combining single-cell omic techniques with other experimental methods such as long-read sequencing. Collectively, these approaches give a more complete look at the molecular landscape of brain cells in neuropsychiatric disorders, paving the way for discovery of biomarkers and therapeutic targets.

### 6.1. Methods of New Single-Cell Omics and Their Application in Postmortem Human Brain

#### 6.1.1. Single-Cell Multi-Omics

Single-cell multi-omics combines two or more single-cell omic modalities. A significant single-cell multi-omic technology is the combined profiling of transcriptome and epigenome in an individual cell. Some versions of single-cell transcriptome plus epigenome profiling rely on physical separation of RNA and DNA for independent analysis, while others rely on separation of reads from RNA and DNA, often by PCR and molecular barcoding. The single-cell capture methods of these technologies include plate- and droplet-based methods.

A prevalent duo includes the profiling of transcriptome and chromatin accessibility, such as scCAT-seq [294], Paired-seq [295], SHARE-seq [296], sci-CAR [297], SNARE-seq/SNARE-seq2 [298,299], ISSAAC-seq [300], and Chromium Epi Multiome ATAC + Gene Expression assay. These methods apply tagmentation of accessible chromatin regions with a transposase, barcoding/indexing of cells, and reverse-transcribing RNA. DNA fragments originating from DNA and reverse-transcribed RNA can be separated for library preparation to then be sequenced. Workflows can target accessible chromatin, chromatin-associated proteins, or their modifications. Indeed, methods have been developed that use specific antibody-conjugated transposases to jointly profile the transcriptome and causes of chromatin accessibility changes, such as histone modifications. Methods that assess both transcriptome and histone modifications include scPCOR-seq [301], CoTECH [265], Paired-Tag [302], droplet-based Paired-Tag [262], EpiDamID [303], SET-seq [304], and scDam&T-seq [305]. Technologies that examine transcriptome and DNA methylome include scM&T-seq [306], scMT-seq [307], and Smart-RRBS [308]. Technologies that examine transcriptome and 3D genomic structure include scCARE-seq [309], Paired Hi-C [274], and MUSIC [170]. There are also single-cell multi-omic methods that assess two epigenetic mechanisms, for example, DNA methylome with chromatin accessibility, including scNOMe-seq [310], scCOOL-seq [311], and iscCOOL-seq [312], or with 3D chromatin conformation such as Methyl-HiC [313] and snm3C-seq [171], and snm3C-seq3 [172]. It is also possible to profile open versus closed chromatin using scGET-seq [314]. Differential enrichment of open and closed chromatin allowed the development of chromatin velocity that provides the trajectories of epigenetic modifications at the single-cell level [314].

Single-cell multi-omics is further capable of assessing more than two omic modalities in one experiment, often profiling the transcriptome along with two epigenomic mechanisms. Transcriptome, chromatin accessibility, and DNA methylome can be profiled together via scNMT-seq [315], scChaRM-seq [316], scNOMeRe-seq [317], and snmCAT-seq [173]. Moreover, TEA-seq [318], DOGMA-seq [319], and NEAT-seq [320] profile transcriptome, chromatin accessibility, and proteins. Other methods, scTrio-seq and scTrio-seq2, assesses transcriptome, along with genomic copy number variations, and DNA methylome [321,322].

#### 6.1.2. Spatial Multi-Omics

Spatial multi-omics provides *spatially resolved* multi-omic information from cells in tissue. These methods can be applied to adjacent tissue sections or on the same tissue by serial (requires retention of analytes) or parallel analyses. Spatial transcriptome and proteome profiling is possible with DBiT-seq [206], CosMx [323], cycleHCR [324], and Spatial-CITE-seq [325]. These technologies can also profile spatial transcriptomics with multiple epigenomic mechanisms, such as Spatial ATAC-RNA-seq [326,327], Spatial CUT&Tag-RNA-seq [326,327], Spatial-DMT [328], Patho-DBiT [329], and SPACE-seq [330].

#### 6.1.3. Single-Cell Multi-Omic Technologies Applied to Postmortem Human Brain

Single-cell multi-omic technologies that have been applied to the human postmortem brain include Chromium Epi Multiome ATAC + Gene Expression [132,137,161,167,168], snm3C-seq [156,171,172], snmC-seq3 [172], MUSIC [170], and snmCAT-seq/snmCT-seq [173,174] (Table 2). Single-cell DNA methylome plus 3D chromatin conformation was profiled in the postmortem human prefrontal cortex of two male donors using snm3C-seq [171]. They found significant cell type-specific associations between DNA methylation and 3D genomic structure, which suggests a high degree of crosstalk between these epigenetic mechanisms [171]. A later study from this group using snm3C-seq simultaneously examined DNA methylation and chromatin conformation in 17 different postmortem human brain regions and observed associations between 3D structural features, mCG, mCH, open chromatin regions, and gene expression [156]. Other studies in postmortem human brain have also found high correlation between gene expression and epigenetic mechanisms [168]. Single-cell multi-omic technologies have also been applied to postmortem human brain for the study of neuropsychiatric disorders, including major depressive disorder, Alzheimer’s disease, Parkinson’s disease, autism spectrum disorder, and post-traumatic stress disorder [132,161,167,170,292] (Table 2 and Table 3). These findings highlight the affluence and diversity of molecular information that can be generated by applying single-cell multi-omic techniques for the study of neuropsychiatric disorders.

#### 6.1.4. Emerging Single-Cell Omics

Single-cell omic technologies have been progressing to explore other omics that have thus far been less prosperous at the single-cell level because of inherent biological constraints. These include single-cell proteomics and metabolomics, the profiling of proteins and metabolites, respectively. Unlike nucleic acids, amino acids cannot be amplified by PCR or IVT; thus, common proteomic techniques such as mass spectrometry (MS) require large amounts of starting material, typically extracted from more than thousands of cells for bulk analyses [331]. Comprehensive and high-throughput proteomic profiling from single cells is technically challenging because of these input requirements, compounded by sample loss during multi-step preparation, and the complexity of protein structures [331]. Metabolites are also structurally complex and dynamic molecules, therefore being difficult to study [332]. As such, MS techniques are also commonly used to analyze metabolites, specifically liquid chromatography-MS (LC/MS) [332].

Technological advancements have been driving proteomics toward a more comprehensive single, cell-level analysis. Improvements to MS, such as orbitrap-based data-independent acquisition (DIA), have increased detection sensitivity for lower input samples [333]. Building on this, Chip-Tip can achieve the quantification of over 5000 proteins per single cell, marking a major leap in proteome coverage [334]. Improved sample preparation methodology, such as nanoPOTS, reduces protein loss [335]. Multiplexed labeling strategies, such as tandem mass tags combined with carrier proteomes in Single Cell ProtEomics by Mass Spectrometry (SCoPE-MS) and SCoPE2, enhanced peptide detection from single cells by boosting the signal and increasing cost-effectiveness, scalability, and ease of use [336,337,338]. However, these techniques often have low cellular throughput [333]. Cellular throughput was increased by CITE-seq, which profiles transcriptome and proteome by NGS, therefore also being multi-omic in nature [339]. CITE-seq uses oligonucleotides, containing a barcode for antibody identification and a PCR handle, that are conjugated with an antibody [339]. These oligonucleotides can be captured by oligo(dT) primers used in single-cell technologies such as Drop-seq for library preparation and NGS [339].

Many single-cell proteomics approaches are compatible with spatial assays and metabolomics because of the antibody-based labeling chemistries. This compatibility has led to the development of spatial proteomics and metabolomics, such as Imaging Mass Cytometry (IMC) [340] and multiplexed ion beam imaging (MIBI) [341]. A 3D IMC has also been developed for single-cell level tissue organization in the original 3D context [342]. These methods can only target predefined proteins. Mass spectrometry (MS)-based imaging methods offer a label-free alternative that can detect a broad range of proteins and metabolites in an unbiased manner. Furthermore, coupling ultra-high sensitivity MS with laser capture microdissection (LCM) in Deep Visual Proteomics (DVP) enables the characterization of histologically defined regions and individual cells [343]. To improve cellular resolution, matrix-assisted laser desorption/ionization mass spectrometry imaging (MALDI-MSI) [344], MALDI-2 [345], and MALDI-MSI in transmission-mode geometry (t-MALDI-MSI) are used [346]. Recent work combined MALDI-MSI for metabolite detection with IMC for cellular phenotyping on the same tissue section [347]. Combining MADI-MSI with the Visium platform, spatial multimodal analysis (SMA), provides spatial resolution of the transcriptome, proteome, and metabolomes [221]. Single-cell live imaging with mass spectrometry (SCLIMS) can simultaneously profile metabolic features and cellular identity at single-cell resolution to inform associations between metabolome and cellular phenotypic features [348]. High-resolution MS (HRMS) provides subcellular resolution of proteomic and metabolomic details [349].

#### 6.1.5. Emerging Single-Cell Omics Combined with Other Techniques Applied to Postmortem Human Brain

Single-cell proteomic and metabolomic methods are relatively new to the field and have therefore not been broadly applied in the human brain. MADI-MSI combined with Visium, SMA, was applied to postmortem caudate/putamen tissue from one male donor with Parkinson’s disease to gain spatial transcriptomic, proteomic, and metabolomic information [221] (Table 3). Additionally, GeoMx DSP has spatially profiled proteins in postmortem human brain tissue, studying autism spectrum disorder, Parkinson’s disease, and Alzheimer’s disease [141,216,217] (Table 3). These methods, SMA and GeoMx DSP, are multi-omic (Table 3). These studies demonstrates the feasibility of using single-cell proteomic and metabolomic technologies for the study of neuropsychiatric disorders in postmortem human brain [221].

#### 6.1.6. Single-Cell Omics Combined with Other Techniques

Another direction that the field of single-cell omics is moving towards is combining single-cell omic technologies with other experimental methodologies such as expansion microscopy [350,351] and long-read sequencing.

#### 6.1.7. Expansion Microscopy

Combining the imaging-based spatial transcriptomic method MERFISH with expansion microscopy [352] reduces the overlap of RNAs by physically expanding the sample for imaging, thus resulting in better detection, resolution, and precise localization of RNA [350]. The previously described Epigenomic MERFISH assay is also compatible with expansion microscopy [289]. Indeed, expansion microscopy for epigenetics (ExEpi) has been developed for the study of spatial epigenomics [353] and single-cell evaluation of post-translational epigenetic encoding (SCEPTRE) [354].

#### 6.1.8. Long-Read/Third-Generation Sequencing

Many single-cell omic techniques currently use NGS, which is limited to short reads that are generally around 300 bp and no longer than about 600 bp. Long-read sequencing (also known as third-generation sequencing) can provide information that is lost from fragmenting transcripts for sequencing. DNA fragmentation is not required for long-read sequencing technologies such as Oxford Nanopore Technologies (ONT) and PacBio single-molecule real-time (SMRT) sequencing. These approaches can sequence DNA fragments that are 10 kb and longer. Previously discussed single-cell methods are often constrained by the read length requirements of NGS, restricting transcript length, detection of splicing events, and transcript isoforms. Single-cell long-read sequencing methods have been developed to overcome these challenges: MAS-ISO-seq [355], ScISOr-seq/seq2 [148,356], SnISOr-seq/seq2 [146,147], SnISOr-ATAC [169], and ScNaUmi-seq [357] for sequencing via PacBio and ONT.

#### 6.1.9. Single-Cell Omics Combined with Other Techniques Applied to Postmortem Human Brain

To our knowledge, single-cell omics combined with expansion microscopy has not yet been applied in postmortem human brain tissue. However, the expansion microscopy technology, dEx-Path, has been applied to human brain pathology samples [358]. The single-cell long-read sequencing method SnISOr-seq has been applied in the postmortem human mid-frontal cortex of two male donors with no preexisting neurodegenerative or neurological disease [146] (Table 2). SnISOr-seq increases exon-spanning long reads, and this study found that exons associated with autism show coordinated and cell type-specific inclusion [146]. The hippocampus of six donors (three male, three female) was used for SnISOr-seq discovering that splicing, transcription start sites, and polyadenylation sites vary extensively between cell types and are associated with disease-linked variations [148] (Table 2). Recently, SnISOr-seq2 was applied to postmortem human brain cortex samples from six donors with frontotemporal dementia (FTD) and six without and found cell type-specific splicing changes [147] (Table 2). SnISOr-ATAC was also applied to nine prefrontal cortex samples from donors with Alzheimer’s disease and ten without to profile chromatin accessibility and splicing patterns [169] (Table 2). These studies demonstrate the benefits of gaining additional biological information from combining other techniques with single-cell omics in neuropsychiatric research.

In conclusion, we have outlined the fundamental methodologies for applying single-cell omic technologies in archival, frozen postmortem human brain tissue for the investigation of neuropsychiatric disorders. There are many avenues for the current and emerging single-cell omic technologies and present or potential application in future neuropsychiatric research. Overall, this technology-centered review provides guidance and resources to inform and inspire future neuropsychiatric research using single-cell omics.

## Data Availability

No new data were created or analyzed in this study. Data sharing is not applicable to this article.
